# Bicarbazole-Benzophenone-Based Twisted Donor-Acceptor-Donor Derivatives as Blue Emitters for Highly Efficient Fluorescent Organic Light-Emitting Diodes

**DOI:** 10.3390/nano14020146

**Published:** 2024-01-09

**Authors:** Dovydas Blazevicius, Iram Siddiqui, Prakalp Gautam, Gintare Krucaite, Daiva Tavgeniene, Mangey Ram Nagar, Krishan Kumar, Subrata Banik, Jwo-Huei Jou, Saulius Grigalevicius

**Affiliations:** 1 Department of Polymer Chemistry and Technology, Kaunas University of Technology, Radvilenu Plentas 19, LT50254 Kaunas, Lithuania; 2 Department of Materials Science and Engineering, National Tsing Hua University, No. 101, Section 2, Guangfu Rd., East District, Hsinchu 30013, Taiwan; 3 School of Chemical Sciences, Indian Institute of Technology—Mandi, Kamand 175005, Himachal Pradesh, India; 4 Department of Chemistry, School of Chemical and Biotechnology, SASTRA Deemed University, Thanjavur 613401, Tamil Nadu, India

**Keywords:** donor-acceptor-donor (D-A-D) derivatives, blue organic light-emitting diode (OLED), high efficiency, blue emission, thermal analysis

## Abstract

This paper delves into the development of a group of twisted donor-acceptor-donor (D-A-D) derivatives incorporating bicarbazole as electron donor and benzophenone as electron acceptor for potential use as blue emitters in OLEDs. The derivatives were synthesized in a reaction of 4,4′-difluorobenzophenone with various 9-alkyl-9′H-3,3′-bicarbazoles. The materials, namely, **DB14**, **DB23**, and **DB29**, were designed with different alkyl side chains to enhance their solubility and film-forming properties of layers formed using the spin-coating from solution method. The new materials demonstrate high thermal stabilities with decomposition temperatures >383 °C, glass transition temperatures in the range of 95–145 °C, high blue photoluminescence quantum yields (>52%), and short decay times, which range in nanoseconds. Due to their characteristics, the derivatives were used as blue emitters in OLED devices. Some of the OLEDs incorporating the **DB23** emitter demonstrated a high external quantum efficiency (EQE_max_) of 5.3%, which is very similar to the theoretical limit of the first-generation devices.

## 1. Introduction

Organic light-emitting diodes (OLEDs) have ushered in a technological revolution with their remarkable impact on diverse sectors of our daily lives. These versatile devices, known for their outstanding display and lighting capabilities, have become indispensable components in different technologies including smartphones, tablets, televisions, and automotive applications [1,2,3,4,5,6]. OLEDs are steadily advancing in terms of their performance, longevity, and production processes [7,8,9]. To provide displays and energy-efficient lighting solutions, blue OLEDs are an essential technological component. Despite their significance, the development of blue OLEDs has posed persistent challenges owing to the inherent instability of blue-emitting materials [10,11,12]. Recent research efforts have resulted in notable progress in the domain of promising host derivatives for the blue devices, offering promising avenues to overcome the efficiency limitations and reaching high internal quantum efficiency [13,14,15,16]. Simultaneously, scientists and engineers have made noteworthy strides in the creation of novel organic emitters tailored for blue OLEDs [17,18,19]. These emitters were engineered to deliver high efficiency, exceptional color purity, and a narrow emission bandwidth, addressing the demands of modern OLED technology [20,21,22,23].

OLED technologies have been generally divided into three generations, depending on the characteristics of the used emitting material, which determines the properties of the diode. First-generation devices use fluorescent emitters [24], second-generation devices use phosphorescent emitters, and third-generation OLEDs are from materials which has a thermally activated delayed fluorescence (TADF) effect. Upon electrical excitation, only 25% of formed excitons in first-generation fluorescent OLEDs are singlet and emissive. The other 75% are of triplet multiplicity and are not involved in light emission [25,26]. Recently, we published carbazole-diphenyl sulfone-carbazole (D-A-D) bipolar blue light emitting materials for first-generation OLEDs. One device outperformed others by showing a peak external quantum efficiency (EQE_max_) of 4.0% for deep-blue emission (CIE_y_ = 0.09) [27].

In this research article, we present a group of twisted D-A-D compounds incorporating bicarbazole and benzophenone fragments that have also been developed for potential use as blue-emitting layers for the OLEDs. These derivatives include 4,4′-bis(9′-butyl-[3,3′]-bicarbazol-9-yl)benzophenone (**DB14**), 4,4′-bis(9′-{2-ethylhexyl}-[3,3′]- bicarbazol-9-yl)benzophenone (**DB23**), and 4,4′-bis(9′-octyl-[3,3′]-bicarbazol-9-yl)benzophenone (**DB29**). The compounds were designed with different alkyl side chains, which determine the film-forming and solubility properties of the blue emitters. Some of the OLED devices based on the bicarbazole and benzophenone fragments containing D-A-D emitters demonstrated promising performance as compared with the carbazole-diphenyl sulfone-carbazole-based emitters. Specifically, one device, producing a greenish-blue emission with a CIE_y_ coordinate of (0.22, 0.22), exhibited an EQE_max_ of 5.3%, which is very similar to the theoretical limit of the first-generation devices [10].

## 2. Experimental Section

### 2.1. Instruments

Thermogravimetric analysis (TGA) of all compounds was carried out using a TGAQ50 instrument (Verder Scientific Haan, Haan, Germany). Both TGA and differential scanning calorimetry (DSC) profiles were recorded under a nitrogen atmosphere at a controlled heating rate of 10 °C/min. For differential scanning calorimetry (DSC), measurements were carried out on Bruker Reflex II thermos-system (Bruker, Berlin, Germany). UV–visible spectroscopy was carried out using an HP-8453 diode array spectrometer (Agilent Technology Inc., Hachioji, Tokyo, Japan) to capture the absorption spectra of the compounds. Moreover, the Tauc plot was generated using the absorbance wavelength data. Photoluminescence (PL) spectra were captured utilizing the Aminco-Bowman Series 2 luminescence spectrometer (Agilent Technology Inc., Hachioji, Tokyo, Japan). For low-temperature PL (LTPL), a Hitachi F-7000 fluorescence spectrophotometer (Hitachi High-Tech, Tokyo, Japan) was employed. The LTPL measurements were performed at a temperature of 77 K to ascertain the singlet energy of the compounds. Cyclic voltammetry (CV) experiments were conducted using the CH instrument CH1604A potentiostat (Annatech Co., Ltd., Taipei, Taiwan). Time-resolved photoluminescence measurements were executed using an Edinburgh instrument spectrometer FLS980 (Edinburgh Instruments Ltd., Livingston, UK) to accurately determine the decay time of the compounds.

### 2.2. Synthesis and Structure Characterization of the Materials

Carbazole (**1**), benzylbromide, bromoethane, 1-bromobutane, 1-bromohexane, 2-ethylhexylbromide, 1-bromooctane, ferric chloride (FeCl_3_), potassium hydroxide (KOH), K_2_CO_3_, NaH, sodium sulphate (Na_2_SO_4_), 4,4′-difluorobenzophenone, chloroform, dimethyl formamide (DMF), and tetrahydrofuran (THF) were purchased from Aldrich and used as received.

3,3′-Bicarbazole (**2**) was prepared by oxidation of carbazole using FeCl_3_ as it was reported in the literature [28].

9-Butyl-9′H-3,3′-bicarbazole (**3**): 1-bromobutane (0.82 g, 6.0 mmol) was added to a stirred solution of 3,3′-bicarbazole (**2**) (2.00 g, 6.0 mmol) in 50 mL of tetrahydrofuran. The reaction mixture was refluxed, and then potassium carbonate (1.66 g, 12.0 mmol) and powdered KOH (2.02 g, 36.1 mmol) were added stepwise. The resulting mixture reacted for 4 h. After TLC test, the inorganic materials were filtered off and the organic derivatives were purified using silica gel column chromatography. A mixture of tetrahydrofuran and hexane (vol. ratio 1:5) was used as an eluent. Yield: 0.96 g (41%) of yellowish material. ^1^H NMR (400 MHz, CDCl_3_, δ, m.d.): 8.29 (s, 2H), 8.11–8.06 (m, 2H), 7.73–7.67 (m, 2H), 7.41–7.32 (m, 6H), 7.17 (t, 2H, *J* = 7.2 Hz), 4.29 (t, 2H, *J* = 7.2 Hz), 1.79 (quint, 2H, *J* = 7.2 Hz), 1.38–1.29 (m, 2H), 0.87 (t, 3H, *J* = 7.2 Hz). ^13^C NMR (400 MHz, CDCl_3_*,* δ, m.d.): 139.64, 138.56, 133.33, 125.94, 125.86, 125.72, 125.57, 124.00, 123.61, 123.43, 123.08, 120.51, 120.46, 119,51, 119.03, 119.00, 118.92, 118.82, 110.83, 110.77, 108.95, 108.86, 42.99, 31.25, 20.65, 13.97. MS (APCI^+^, 20 V): 339.10 ([M + H], 100%).

9-(2-Ethylhexyl)-9′H-3,3′-bicarbazole (**4**) was prepared by a similar method which was published earlier [27]. 

9-Octyl-9′H-3,3′-bicarbazole (**5**): 1-Bromooctane (1.16 g, 6.0 mmol) was added to a stirred solution of 3,3′-bicarbazole (**2**) (2.00 g, 6.0 mmol) in 50 mL of tetrahydrofuran. The mixture was heated to reflux, and then potassium carbonate (1.66 g, 12.0 mmol) and powdered KOH (2.02 g, 36.2 mmol) were added stepwise. The resulting mixture reacted for 4 h. After TLC test, the inorganic compounds were filtered off and the organic materials were separated using silica gel column chromatography. A mixture of tetrahydrofuran and hexane (vol. ratio 1:7) was an eluent. Yield: 1.15 g (43%) of yellowish material. ^1^H NMR (400 MHz, CDCl_3_, δ, m.d.): 8.45 (d, 2H, *J* = 8.0 Hz), 8.25–8.20 (m, 2H), 8.04 (s, 1H), 7.87 (d, 1H, *J* = 8.0 Hz), 7.83 (dd, 1H, *J*_1_ = 8.4 Hz, *J*_2_ = 1.6 Hz), 7.56–7.50 (m, 3H), 7.48 (d, 3H, *J* = 7.2 Hz), 7.31 (t, 2H, *J* = 8.0 Hz), 4.37 (t, 2H, *J* = 7.0 Hz), 1.95 (qu, 2H, *J* = 7.3 Hz), 1.48–1.30 (m, 10H), 0.92 (t, 3H, *J* = 7.0 Hz). ^13^C NMR (400 MHz, CDCl_3_, δ, m.d.): 140.94, 140.02, 139.62, 138.53, 133.30, 125.94, 125.85, 125.70, 125.56, 124.00, 123.61, 123.42, 123.08, 120.49, 120.45, 120.40, 119.51, 119.00, 118.92, 118.79, 110.80, 110.74, 108.92, 108.83, 43.25, 31.85, 29.45, 29.23, 29.08, 27.39, 22.65, 14.12. MS (APCI^+^, 20 V): 444.67 ([M + H], 100%).

4,4′-Bis(9′-butyl-[3,3′]-bicarbazol-9-yl)benzophenone (**DB14**): 9-Butyl-9′H-3,3′-bicarbazole (**3**) (0.40 g, 1.0 mmol) and NaH (0.10 g, 4.1 mmol) were stirred in 6 mL of DMF at room temperature under nitrogen for 20 min. Then, 4,4′-difluorobenzophenone (0.11 g, 0.5 mmol) was added to the reaction, and the resulting mixture was stirred at 150 °C under nitrogen for 4 h. After TLC control, the reaction mixture was cooled and quenched by the addition of ice water. The product was extracted using chloroform. The combined extract was dried over anhydrous Na_2_SO_4_. The crude product was purified using silica gel column chromatography using the mixture of THF and hexane (vol. ratio 1:3) as an eluent. Yield: 0.38 g (77%) of yellow amorphous material. T_g_ = 145 °C (DSC). ^1^H NMR (400 MHz, CDCl_3_, δ, m.d.): 8.51–8.41 (m, 4H), 8.30–8.23 (m, 6H), 7.91–7.83 (m, 8H), 7.71 (d, 2H, *J* = 8.4 Hz), 7.65 (d, 2H, *J* = 8.4 Hz), 7.57–7.38 (m, 10H), 7.32–7.29 (m, 4H), 4.42–4.36 (m, 4H), 1.98–1.91 (m, 4H), 1.51–1.45 (m, 4H), 1.01 (t, 6H, *J* = 7.4 Hz). MS (APCI^+^, 20 V): 954.8 ([M + H], 100%).

4,4′-Bis(9′-{2-ethylhexyl}-[3,3′]-bicarbazol-9-yl)benzophenone (**DB23**): 9-[2-Ethylhexyl]-9′H-3,3′-bicarbazole (**4**) (0.40 g, 0.9 mmol) and NaH (0.10 g, 4.1 mmol) were stirred in 6 mL of DMF at room temperature under nitrogen for 20 min. Then, 4,4‘-difluorobenzophenone (0.10 g, 0.5 mmol) was added to the reaction, and the resulting mixture was stirred at 150 °C under nitrogen for 4 h. After TLC control, the reaction mixture was cooled and quenched by the addition of ice water. The product was extracted using chloroform. The combined extract was dried over anhydrous Na_2_SO_4_. The crude product was purified using silica gel column chromatography using the mixture of THF and hexane (vol. ratio 1:5) as an eluent. Yield: 0.40 g (83%) of yellow amorphous material. T_g_ = 104 °C (DSC). ^1^H NMR (400 MHz, CDCl_3_-, δ, m.d.): 8.51–8.45 (m, 4H), 8.31–8.22 (m, 6H), 7.91–7.79 (m, 8H), 7.76–7.70 (m, 4H), 7.66–7.36 (m, 14H), 4.27–4.23 (m, 4H), 1.90–1.87 (m, 2H), 1.47–1.29 (m, 16H), 1.00–0.90 (m, 12H). ^13^C NMR (400 MHz, CDCl_3_*,* δ, m.d.): 195.67, 141.42, 140.70, 140.22, 135.79, 132.79, 131.96, 129.44, 128.17, 127.18, 126.36, 126.13, 125.75, 125.49, 125.45, 124.56, 124.19, 123.42, 122.98, 120.77, 120.41, 119.02, 118.92, 118.82, 118.77, 110.08, 109.95, 109.27, 109.13, 47.57, 39.50, 31.06, 28.89, 24.45, 23.10, 14.09, 10.96. MS (APCI^+^, 20 V): 1066.55 ([M + H], 100%). 

4,4′-Bis(9′-octyl-[3,3′]-bicarbazol-9-yl)benzophenone (**DB29**): 9-Octyl-9′H-3,3′-bicarbazole (**5**) (0.40 g, 0.9 mmol) and NaH (0.10 g, 4.1 mmol) were stirred in 6 mL of DMF at room temperature under nitrogen for 20 min. Then, 4,4‘-difluorobenzophenone (0.10 g, 0.5 mmol) was added to the reaction and the resulting mixture was stirred at 150 °C under nitrogen for 4 h. After TLC control, the reaction mixture was cooled and quenched by the addition of ice water. The product was extracted using chloroform. The combined extract was dried over anhydrous Na_2_SO_4_. The crude product was purified using silica gel column chromatography using the mixture of THF and hexane (vol. ratio 1:5) as an eluent. Yield: 0.44 g (92%) of yellow amorphous material. T_g_ = 95 °C (DSC). ^1^H NMR (400 MHz, CDCl_3_-, δ, m.d.): 8.50 (dd, 4H, *J*_1_ = 15.2 Hz, *J*_2_ = 1.6 Hz), 8.30 (d, 2H, *J* = 7.6 Hz), 8.27–8.24 (m, 6H), 7.91–7.86 (m, 8H), 7.71 (d, 2H, *J* = 8.4 Hz), 7.66 (d, 2H, *J* = 8.0 Hz), 7.56–7.47 (m, 8H), 7.42 (t, 2H, *J* = 7.2 Hz), 7.33–7.29 (m, 2H), 4.38 (t, 4H, *J* = 7.2 Hz), 1.99–1.92 (m, 4H), 1.48–1.30 (m, 20H), 0.92 (t, 6H, *J* = 6.6 Hz). ^13^C NMR (400 MHz, CDCl_3_*,* δ, m.d.): 194.51, 142.04, 140.96, 140.71, 139.74, 139.28, 135.78, 135.41, 132.84, 131.97, 126.38, 126.34, 126.15, 125.79, 125.49, 124.57, 124.20, 123.48, 123.03, 120.78, 120.66, 120.50, 119.02, 118.85, 110.09, 109.97, 109.00, 108.87, 43.26, 31.85, 29.45, 29.23, 29.08, 27.39, 22.65, 14.12. MS (APCI^+^, 20 V): 1067.78 ([M + H], 100%).

### 2.3. Device Fabrication

OLED devices were fabricated utilizing a pre-sputtered ITO glass substrate. The substrate underwent a thorough cleaning process, including 30 min treatments with acetone and isopropyl alcohol (IPA) at elevated temperatures of 50 and 60 °C. Subsequently, the substrates were transferred to a UV chamber that was preheated, and exposed to UV light for 10 min. The deposition of layers occurred within a glove box, maintaining an inert atmosphere. The hole injection layer (PEDOT:PSS) was spin-coated onto the substrates for 20 s at 4000 rpm, followed by a 10 min heating step at 130 °C. Subsequently, the cooled substrates were subjected to a 20-s spin-coating process at 2500 rpm for the emissive layer. The subsequent stages encompassed the thermal evaporation of the electron-injecting/transporting layer and aluminum cathode within a vacuum of 1.33 × 10^−4^ Pa. The substrates remained under vacuum conditions within a mini chamber in the glove box to preserve their quality until individual testing. All testing protocols were executed in a completely dark environment under ambient conditions. The CS-100A luminescence spectrophotometer was employed for recording the current density–voltage–luminance (J-V-L) characteristics, while the PR-655 spectrophotometer was used for power efficacy–luminance–current characteristics. A Keithley voltmeter was utilized to measure the current–voltage (I-V) characteristics. The device area was determined to be 0.09 cm^2^. The EQE of the devices was computed following the methodology outlined in the relevant literature, meticulously adhering to this approach during the testing and analysis procedures [20].

## 3. Results and Discussion

**Scheme 1:**
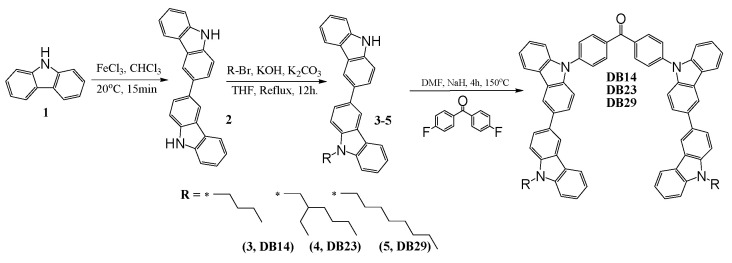
Synthetic pathway of **DB14**, **DB23**, and **DB29**.

A three-step synthetic pathway was employed to synthesize the bicarbazole-based materials, as is demonstrated in Scheme 1.

3,3′-Bicarbazole (**2**) was synthesized through the oxidation of 9H-carbazole (**1**) using iron (III) chloride, as is described in the ESI file. Various 9-alkyl-9′H-3,3′-bicarbazoles (**3**–**5**) were prepared via N-alkylation reactions between a bicarbazole and the corresponding alkyl bromides, utilizing potassium hydroxide and potassium carbonate in tetrahydrofuran (THF). The next step involved the nucleophilic aromatic substitution of partially alkylated bicarbazole with 4,4′-difluorobenzophenone. This reaction was conducted in DMF, employing sodium hydride as a base, yielding the desired materials. This multi-step process resulted in the successful synthesis of the targeted bicarbazole-based compounds. The values of glass transition temperatures are influenced by the distinct alkyl chains in the chemical structures of materials **DB14**, **DB23**, and **DB29**. The derivative **DB14**, characterized by the shortest butyl alkyl chain, exhibited the highest glass transition temperature at 145 °C. The elongation of the alkyl chain led to decreased but still suitable T_g_ values for device forming, with **DB23** and **DB29** showing temperatures of 105 °C and 95 °C, respectively, as verified by DSC.

### 3.1. Material Characteristics

#### 3.1.1. DFT Calculations

**Figure 1:**
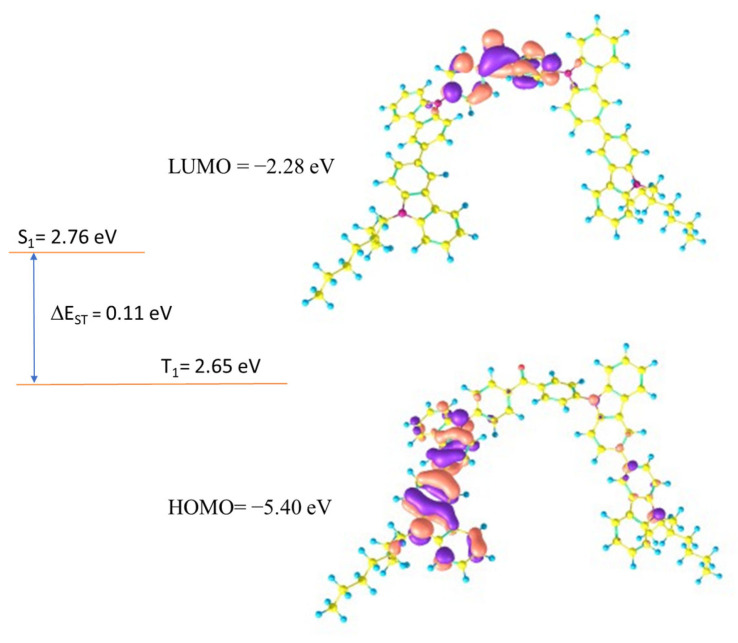
Electron density contours of FMO and theoretically calculated electronic properties of the compound **DB23**.

Theoretical calculations were carried out to establish a relation between the electrochemical properties and geometries of the synthesized compounds. All calculations were carried out using Gaussian09.0D [29]. The polarizable continuum model, which Gaussian09 uses by default, was employed for the solvent adjustments [30]. In all compounds, HOMO was found to be located over the peripheral carbazole units, while LUMO was found to be spread over the central benzophenone unit. The HOMO/LUMO distributions ensure that the present molecules have donor-acceptor characteristics, in which carbazole units act as donor units and benzophenone acts as an acceptor unit. The values of HOMO/LUMO were theoretically calculated to be −5.39/−2.28, −5.40/−2.28, and −5.40/−2.26 eV for **DB14**, **DB23**, and **DB29**, respectively. Further, the singlet and triplet energies were very crucial for the designed emitters to evaluate their potential for optoelectronic devices; hence, the singlet and triplet energy levels of all synthesized compounds were established through TD-DFT calculations. All compounds showed high triplet energies of about 2.65 eV, which indicated that these molecules have the potential to be used in the emitting layer of OLEDs. The HOMO/LUMO and singlet/triplet energies of **DB23** are shown in Figure 1. Similarly, the theoretical results for **DB13** and **DB29** are presented in Figure S1 of ESI. All theoretical results are also tabulated in Table 1.

#### 3.1.2. Photophysical Properties

**Figure 2:**
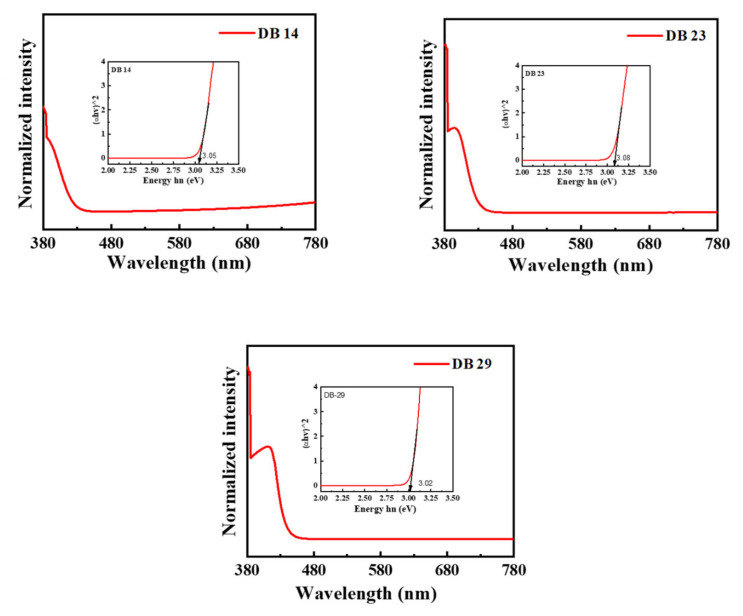
Ultraviolet-visible absorbance spectra and Tauc plots (inset) of the compounds **DB14**, **DB23**, and **DB29**.

The derivatives **DB14**, **DB23**, and **DB29** exhibit substantial photoluminescence quantum yields (PLQY) of 55.6, 52.0, and 55.5%, respectively. The PLQY values are summarized in Table 1. The UV-absorption bands of these compounds are demonstrated in Figure 2. The materials were investigated using tetrahydrofuran (THF) solvent under typical conditions. Notably, all compounds displayed primary and secondary absorption peaks consistently at around 375 and 400 nm. The UV absorption wavelength and intensity were also used to construct the Tauc plots for the materials (Figure 2) using the following values: (α × hν)^1/2^ for the x-axis and hν for the y-axis, where α is the intensity and hν is the energy (hν = 1240/wavelength). The Tauc plots demonstrated the bandgaps of the investigated compounds, which are 3.05 eV for **DB14**, 3.08 eV for **DB23**, and 3.02 eV for **DB29** (Table 1).

**Figure 3:**
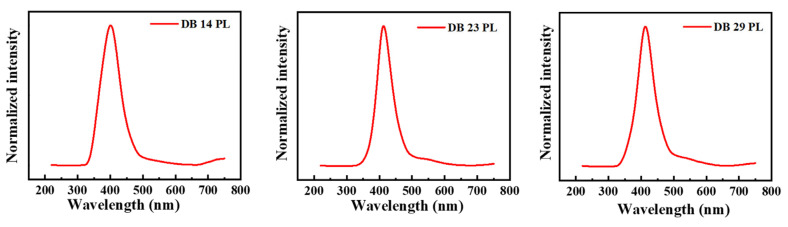
Photoluminescence (PL) spectra of the derivatives **DB14**, **DB23**, and **DB29**.

For the measurements of photoluminescence, the maxima absorbance wavelengths were employed as the wavelengths for excitation, which are shown in Table 1. In Figure 3, the photoluminescence (PL) spectra of the derivatives **DB14**, **DB23**, and **DB29** are depicted. They show very similar emission bands for all compounds with maxima wavelengths of approximately 400 nm due to the similar electronic structure of the derivatives.

**Figure 4:**
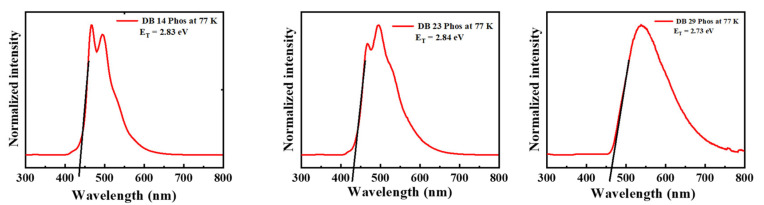
LTPL spectra of the derivatives **DB14**, **DB23**, and **DB29** at 77 K of the compounds.

Moreover, to establish the triplet energies of the potential emitters, photoluminescence (LTPL) spectra at low temperature were also recorded. Notably, the compounds **DB14**, **DB23**, and **DB29** exhibited elevated triplet energies of 2.83, 2.84, and 2.73 eV, respectively, as visually represented in Figure 4. These specific characteristics are also included in Table 1 for reference.

**Figure 5:**
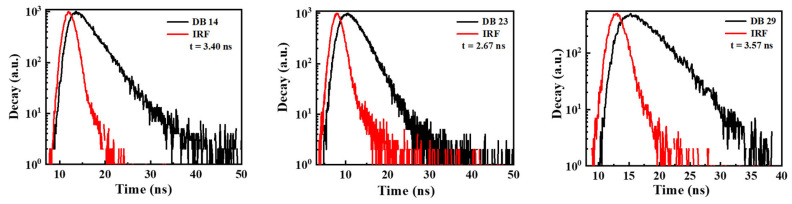
TRPL spectra for the transient decay of the materials **DB14**, **DB23**, and **DB29**. IRF is the instrument response function, which is measured as a control parameter before and after each measurement.

In Figure 5, the time-resolved photoluminescence (TRPL) analysis illustrates the values of the decay times for the **DB14**, **DB23**, and **DB29** compounds, which were 3.40, 2.67, and 3.57 ns, respectively. It could be stated that all lifetime curves of the materials demonstrate a nanosecond-scale component. Usually, the decay lifetime of fluorescent emitters ranges in picoseconds. However, our materials demonstrate decay in the nanoscale, also indicating a possible use of triplet energy levels.

#### 3.1.3. Electrochemical Properties

**Figure 6:**
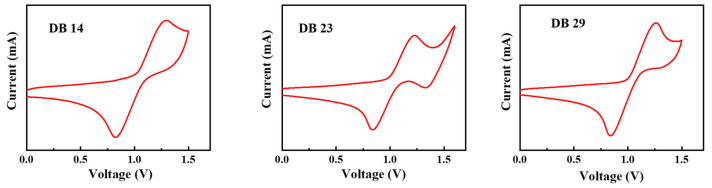
Curves of CV scans of the molecules **DB14**, **DB23**, and **DB29**.

The electrochemical attributes of the **DB14**, **DB23,** and **DB29** compounds were assessed through cyclic voltammetry (CV) measurements, which are depicted in Figure 6. By using the obtained oxidation onset values, the HOMO levels were calculated using Equation (1) and the LUMO levels were calculated using Equation (2), according to the method described in the literature [31,32]:

(1) $$E_{\mathrm{HOMO}}=-[4.4+E_{onset}^{ox}]$$

**E_LUMO_** = **E_HOMO_** + **E_g_** (2)

The calculated HOMO levels were −5.70, −5.63, and −5.66 eV, while the corresponding LUMO levels were −2.65, −2.55, and −2.64 eV for the **DB14**, **DB23**, and **DB29** compounds, respectively. These values along with the earlier bandgap measurements are presented in Table 1. It could be stated that the HOMO and LUMO levels of the compounds are found to be suitable for blue-emitting layers using the earlier-mentioned CBP host material.

#### 3.1.4. Thermal Properties

**Figure 7:**
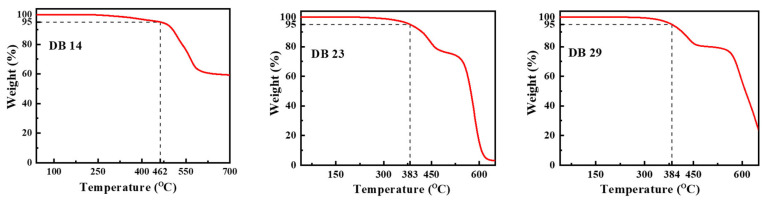
Curves of the TGA measurements of the materials **DB14**, **DB23**, and **DB29**.

The characteristics of thermal stability of the materials **DB14**, **DB23**, and **DB29** were examined in a nitrogen atmosphere. The curves of the thermogravimetric analysis (TGA) are presented in Figure 7. For **DB14**, having the shortest alkyl chains, the temperature at which 5% weight loss occurred, i.e., the decomposition temperature (T_d_), was obtained at 462 °C during heating. The materials **DB23** and **DB29**, featuring lengthier aliphatic substitutions, exhibited a lower thermal stability, with T_d_ values of 383 °C and 384 °C, respectively. These findings demonstrate that the new materials have very good thermal stability as emitting materials for application in OLED devices.

**Figure 8:**
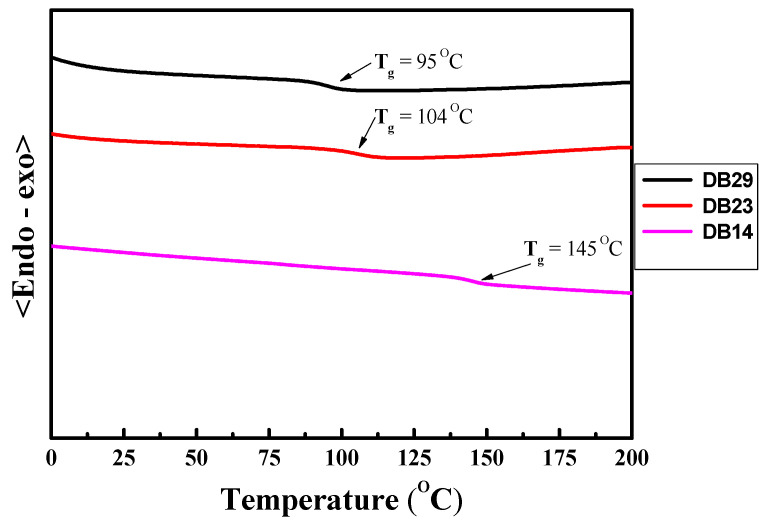
DSC curves of second heating of the materials **DB14**, **DB23**, and **DB29**.

The DSC thermograms of second heating for the **DB14**, **DB23**, and **DB29** compounds are displayed in Figure 8. Notably, upon analyzing the second heating curves, it becomes evident that the novel derivatives exhibit glass transition temperatures (T_g_) that directly correlate with the length of their alkyl substituents. For instance, the **DB14** material with the shortest butyl substitution has the highest glass transition temperature of 145 °C. This trend persists also for compounds featuring more extended alkyl groups: the **DB23** and **DB29** derivatives with 2-ethylhexyl and octyl substitutions, respectively, display glass transition temperatures of 104 °C and 95 °C. Hence, these results indicated that the developed compounds demonstrate a high thermal stability and are suitable for optoelectronic devices. The comprehensive T_d_ and glass transition temperature (T_g_) values are compiled in Table 1.

#### 3.1.5. Electroluminescent Properties

**Figure 9:**
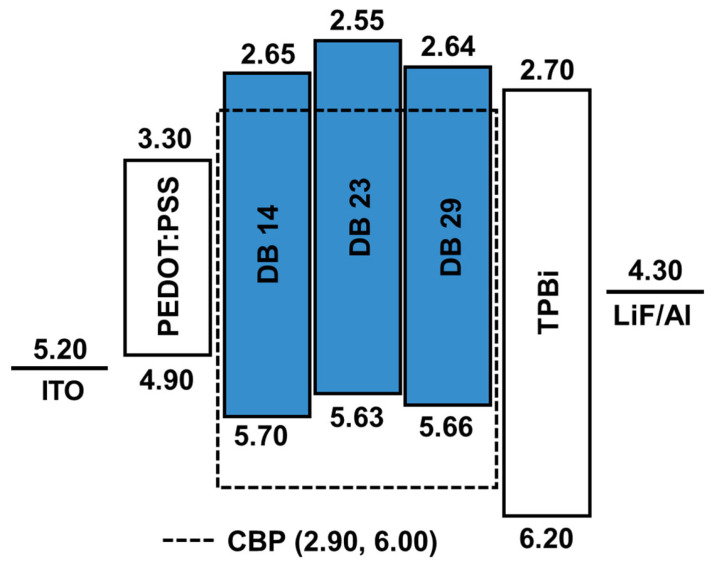
Energy-level diagram of the OLEDs using the emitters **DB14**, **DB23**, and **DB29** doped in CBP host.

The schematic energy level diagram in Figure 9 illustrates the configuration of OLED devices prepared in this study. These devices incorporated the emitters **DB14**, **DB23**, and **DB29** doped within a CBP host matrix. The device structure comprised ITO/PEDOT:PSS/host CBP: emitter **DB14**, **DB23**, or **DB29** (5, 10, or 15wt.%)/TPBi/LiF/Al.

**Figure 10:**
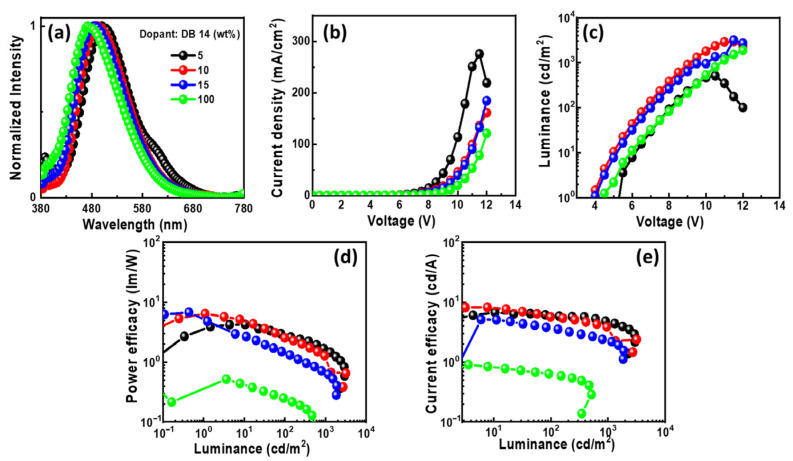
EL properties of the devices using the emitter **DB14** doped in CBP host: (**a**) EL spectra, (**b**) current density–voltage, (**c**) luminance–voltage, (**d**) power efficacy–luminance, and (**e**) current efficacy–luminance characteristics.

**Figure 11:**
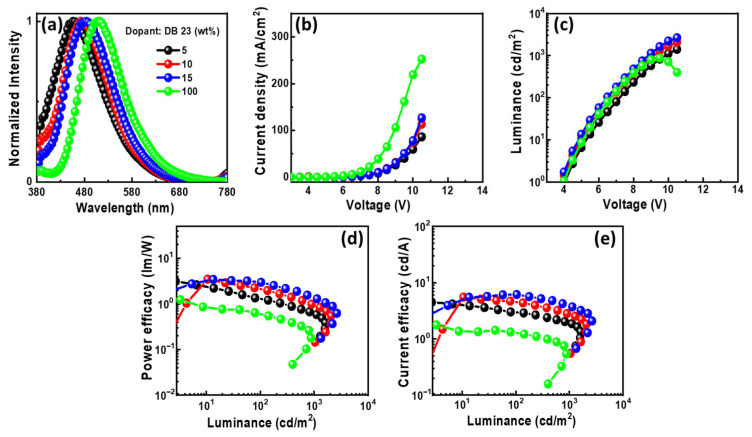
The EL properties of the OLEDs using emitting material **DB23** doped in CBP host: (**a**) EL spectra, (**b**) current density–voltage, (**c**) luminance–voltage, (**d**) power efficacy–luminance, and (**e**) current efficacy–luminance characteristics.

**Figure 12:**
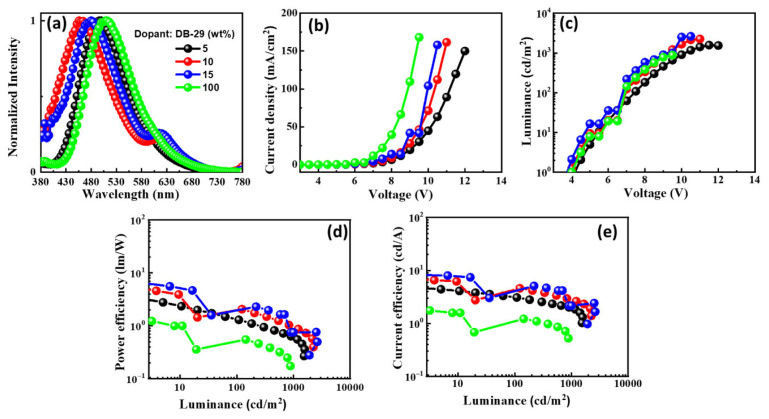
The EL characteristics of the OLEDs with emitter **DB29** doped in CBP host material: (**a**) EL spectra, (**b**) current density–voltage, (**c**) luminance–voltage, (**d**) power efficacy–luminance, and (**e**) current efficacy–luminance characteristics.

The electroluminescence (EL) properties of the devices using the new emitting materials dispersed within the CBP host are shown in Figure 10, Figure 11 and Figure 12. The corresponding characteristics for each emitter are also detailed in Table 2. Within each figure, various aspects are illustrated: (a) EL spectra, (b) current density–voltage characteristics, (c) luminance–voltage characteristics, (d) power efficacy–luminance characteristics, and € current efficacy–luminance characteristics.

In Figure 10a, the EL spectra of the OLEDs with **DB14** exhibit peaks around 480 nm, indicating blue emission. The presence of sole peaks implies the successful energy transfer between host and guest. The emission wavelength in the EL of the devices undergoes a shift when compared with the PL spectra of the **DB14** emitter, indicating the influence of the CBP host on emission. An evident emission spectrum of the emitter becomes apparent with an increase in the concentration of the **DB14** dopant. Notably, both non-doped and doped devices exhibit very similar EL emission peaks. In Figure 10b–e, the current density–luminance–voltage and power efficacy–luminance–current efficacy characteristics are depicted. The non-doped device exhibits a significantly lower efficacy compared to the doped devices, highlighting the substantial role of the host material. Consequently, a device based on a 10 wt% doping concentration surpasses others in terms of power efficacy, showcasing a PE_max_ of 4.4 lm/W, a CE_max_ of 7.6 cd/A, an EQE_max_ of 3.3%, and an L_max_ of 3175 cd/m^2^ at a turn-on voltage of 5.1 V.

In Figure 11a, the EL spectra of the devices with **DB23** exhibit single peaks at around 470 nm, indicating blue emission. The presence of single peaks suggests the successful host–guest energy transfer completion. The emission wavelength in the EL of the devices undergoes a shift when compared with the PL spectra of the **DB23** emitter, indicating the influence of the CBP host on emission. An evident emission spectrum of the emitter becomes apparent with an increase in the concentration of the **DB23** dopant. Notably, both doped and non-doped devices exhibit similar EL emission peaks. Figure 11b–e demonstrate current density–luminance–voltage and power efficacy–luminance–current efficacy characteristics. Comparatively, the non-doped device shows a considerably lower efficacy than the doped devices, demonstrating the crucial role of the host material. The device utilizing a 15 wt% doping concentration of the emitter has a maxima PE_max_ of 3.2 lm/W, a CE_max_ of 5.3 cd/A, and an L_max_ of 2076 cd/m^2^ at a voltage of 5.1 V. Additionally, the device having a 10 wt% doping concentration reveals a remarkable EQE_max_ of 5.3%, even surpassing the theoretical limit for fluorescent emitters. The emitter **DB23**-based device stands out in comparison to the others.

The EL spectra of the **DB29**-based device exhibit a blue shift with increasing doping concentration, in contrast to the non-doped device, as can be seen in Figure 12a. The EL spectra maxima change from approximately 490 nm to 430 nm in the region of blue emission. Single peaks of the emissions signify the achievement of complete energy transfer between host and guest. The emission wavelength in the EL of the devices undergoes a shift when compared with the PL spectra of the **DB29** emitter, indicating the influence of the CBP host on emission. An evident emission spectrum of the emitter becomes apparent with an increase in the concentration of the **DB29** dopant. Figure 12b–e illustrate the current density–luminance–voltage and power efficacy–luminance–current efficacy characteristics. The non-doped device showcases a more favorable current density–voltage characteristic compared to the doped devices, although its efficacy is considerably lower than that of its doped counterparts. Consequently, the role of the host material is pronounced, leading to the prominence of the device with a 15 wt% doping concentration. This device outperforms others in terms of power efficacy, exhibiting a PE_max_ of 7.9 lm/W, a CE_max_ of 9.1 cd/A, an EQE_max_ of 4.0%, and an L_max_ of 2631 cd/m^2^ at a voltage of 4.7 V. Impressively, this device showcases the highest PE and CE values among all.

As depicted in Table 2, the **DB23**-based device exhibits the highest EQE among all devices, surpassing both previous research and the outcomes of this study. This enhanced performance could be attributed to the inclusion of the branched alkyl side chains in the molecule, which likely contributed to improved molecule solubility for the fabrication of wet-processed OLED devices and the film-forming properties of the material. Nevertheless, the PE and CE of the **DB29**-based device demonstrated superior performance. This can be attributed to its appropriate HOMO and LUMO levels, which facilitated the efficient host–guest energy transfer. Additionally, the incorporation of two donor moieties of the bicarbazole contributed to a balanced charge transfer, further enhancing its performance.

## 4. Conclusions

In conclusion, this study presents an exploration of twisted donor-acceptor-donor compounds, specifically bicarbazole-benzophenone-bicarbazole-based compounds, as potential blue emitters for blue OLEDs. The synthesized emitters **DB14**, **DB23**, and **DB29**, were systematically designed with tailored alkyl side chains to enhance their film-forming and solubility properties. The investigation encompassed thorough characterization, including photo-physical analysis to determine photoluminescence quantum yields, UV absorption, and emission characteristics. Electrochemical measurements provided insights into HOMO and LUMO energy levels, while a thermal analysis unveiled the remarkable thermal stability of the compounds. The OLED devices incorporating these emitters demonstrated noteworthy performance, with **DB23** exhibiting the highest EQE among all devices, reaching a peak value of 5.3%. The study also highlighted the significant influence of host–guest energy transfer, optimal doping concentrations, and molecular structures on device efficiency. Notably, the research outcomes underscore the considerable potential of the newly developed D-A-D derivatives in advancing OLED technology. This work contributes to the ongoing progress of OLED materials and their applications, offering insights into the design of efficient blue emitters. This research not only contributes to the understanding of advanced OLED materials but also provides insights into designing efficient blue emitters for future display and lighting applications, thereby advancing the field of organic electronics.

## Figures and Tables

**Table 1:** Optoelectronic and thermal characteristics of the derivatives **DB14**, **DB23**, and **DB29**.

Emitter	λ_ex_ (nm)	λ_em_ (nm)	E_g_ (eV)	Φ (%)	Decay (ns)	Homo (eV)		Lumo (eV)		S_1_ (eV)		T_1_ (eV)		ΔE_ST_		T_d_ (°C)	T_g_ (°C)
						Th.	Ca.	Th.	Ca.	Th.	Ca.	Th.	Ca.	Th.	Ca.		
DB14	366.7, 383.2	401.7	3.05	55.6	3.40	−5.39	−5.70	−2.28	−2.65	2.76	3.32	2.65	2.83	0.11	0.49	462	145
DB23	380.0, 393.8	412.8	3.08	52.0	2.67	−5.40	−5.63	−2.28	−2.55	2.76	3.19	2.65	2.84	0.11	0.35	383	104
DB29	382.7, 411.7	413.3	3.02	55.5	3.57	−5.40	−5.66	−2.26	−2.64	2.78	3.22	2.66	2.73	0.12	0.49	384	95

λ_ex_: excitation wavelength; λ_em_: emission wavelength; E_g_: bandgap; Φ: photoluminescence yield; S_1_: singlet energy; T_1_: triplet energy; ΔE_ST_: singlet-triplet energy gap; T_d_: decomposition temperature; T_g_: glass transition temperature; Th.: theoretical value; Ca.: calculated value.

**Table 2:** EL properties of the OLEDs with the emitting materials **DB14, DB29,** and **DB23** doped in CBP host matrix.

Emitter	Concentration of Emitter in the Host Material (wt%)	Turn-On Voltage (V_on_) ^a^	Power Efficacy (lm/W)	Current Efficacy (cd/A)	EQE (%)	CIE	Max Luminance (cd/m^2^)
			@100/1000 cd/m^2^ and Max				
DB-14	5.0	5.0	2.9/1.6/3.9	6.1/4.7/6.5	2.6/2.2/2.7	(0.22, 0.36)/(0.21, 0.32)/-	2951
	10	5.1	2.6/1.3/4.4	5.7/3.9/7.6	2.6/2.0/3.3	(0.21, 0.33)/(0.20, 0.29)/-	3175
	15	5.9	1.4/1.3/2.4	3.7/2.3/4.9	1.9/1.4/2.4	(0.20, 0.28)/(0.19, 0.24)/-	1884
	100	6.1	0.2/-/0.4	0.6/-/0.8	0.3/-/0.4	(0.26, 0.40)/-	515
DB-29	5.0	5.4	0.3/0.6/2.1	3.1/2.0/3.9	2.0/1.4/2.3	(0.19, 0.22)/(0.22, 0.22)/-	1578
	10	5.7	1.7/1.0/4.4	3.7/2.8/6.5	2.0/1.7/3.1	(0.20, 0.27)/(0.23, 0.25)/-	2251
	15	4.7	1.0/0.3/7.9	2.3/1.0/9.1	1.1/0.5/4.0	(0.21, 0.31)/(0.22, 0.27)/-	2631
	100	5.8	0.4/-/1.4	0.8/-/1.8	0.3/-/0.7	(0.26, 0.44)/-	884
DB-23	5.0	5.7	1.3/0.6/2.1	3.0/1.9/3.8	1.9/1.4/2.3	(0.17, 0.07)	883
	**10**	**5.2**	**1.4/0.8/1.7**	**2.4/1.7/2.6**	**5.1/3.5/5.3**	**(0.19, 0.22)/(0.22, 0.22)/-**	**1620**
	15	5.2	2.2/1.0/3.2	4.6/2.8/5.3	2.5/1.7/2.7	(0.20, 0.27)/(0.23, 0.25)/-	2076
	100	5.1	0.6/-/1.0	1.3/-/1.8	0.5/-/0.7	(0.26, 0.44)/-	875

^a^ Turn-on voltage at luminance >1 cd m^2^.

## Data Availability

The data presented in this study are available on request from the corresponding authors.

## Supplementary Materials

The following supporting information can be downloaded at: https://www.mdpi.com/article/10.3390/nano14020146/s1.
