# Supplementary material for: Bicarbazole-Benzophenone-Based Twisted Donor-Acceptor-Donor Derivatives as Blue Emitters for Highly Efficient Fluorescent Organic Light-Emitting Diodes
Source: Nanomaterials (Basel). 2024 Jan 9;14(2):146. doi: 10.3390/nano14020146 (PMC10819850; doi:10.3390/nano14020146)
Supplement: Supplementary file 1 [file nanomaterials-14-00146-s001.zip › nanomaterials-2727582-supplementary.pdf]

Electronic supporting information

Bicarbazole-benzophenone Based Twisted D-A-D Derivatives as Blue Emitters for Highly Efficient Fluorescent OLEDs

Dovydas Blazevicius[1, §], Iram Siddiqui[2, §], Prakalp Gautam[2], Gintare Krucaite[1], Daiva Tavgeniene[1], Mangey Ram Nagar[2], Krishan Kumar[3], Subrata Banik[4], Jwo-Huei Jou[2*] and Saulius Grigalevicius[1*]

[1]Department of Polymer Chemistry and Technology, Kaunas University of Technology, Radvilenu Plentas 19, LT50254 Kaunas, Lithuania

[2]Department of Materials Science and Engineering, National Tsing Hua University, No. 101, Section 2, Guangfu Rd., East District, Hsinchu, Taiwan, 30013

[3]School of Chemical Sciences, IIT Mandi, Himachal Pradesh 175005, India

[4]Department of Chemistry, School of Chemical and Biotechnology, SASTRA Deemed University, Thanjavur 613401, Tamil Nadu, India.

[§]These authors contributed equally to this work.

Corresponding authors e-mail: saulius.grigalevicius@ktu.lt and jjou@mx.nthu.edu.tw

Synthesis of 9H,9′H-[3,3′]bicarbazole

9H,9′H-[3,3′]bicarbazole was obtained by chemical oxidation of 9H-carbazole in the presence of $FeCl_3$. Anhydrous $FeCl_3$ (20 g, 120 mmol) was added to a magnetically stirred solution of 9H-carbazole (5 g, 30 mmol) in 100 ml of chloroform. The mixture was stirred for 30 min and poured into methyl alcohol. The precipitated material was recovered by filtration and the crude product was purified by silica gel column chromatography using hexane/ ethylacetate (vol. ratio 1:1) as an eluent. The yield of the 9H,9′H-[3,3′]bicarbazole was 62 % (3.13 g). MS($APCI^+$, 20 V), m/z(%): 334 ($[M+H]^+$, 95). ^1H NMR spectrum (DMSO, δ, ppm): 11.31 (s, 2H, NH), 8.55 (s, 2H), 8.27 (d, 2H, *J*=7.8 Hz), 7.84 (d, 2H, *J*=9 Hz), 7.62 (d, 2H, *J*=8.4 Hz), 7.55 (d, 2H, *J*=7.8 Hz), 7.46-7.4 (m, 2H), 7.25-7.18 (m, 2H). IR (KBr): 3419, 3048, 1604, 1491, 1468, 1456, 1241, 753. Elemental analysis for $C_{24}H_{16}N_2$: % Calc. N 8.43, C 86.72, H 4.85; % Found N 8.52, C 86.77, H 4.82.

Results of DFT Calculations of DB14 and DB29

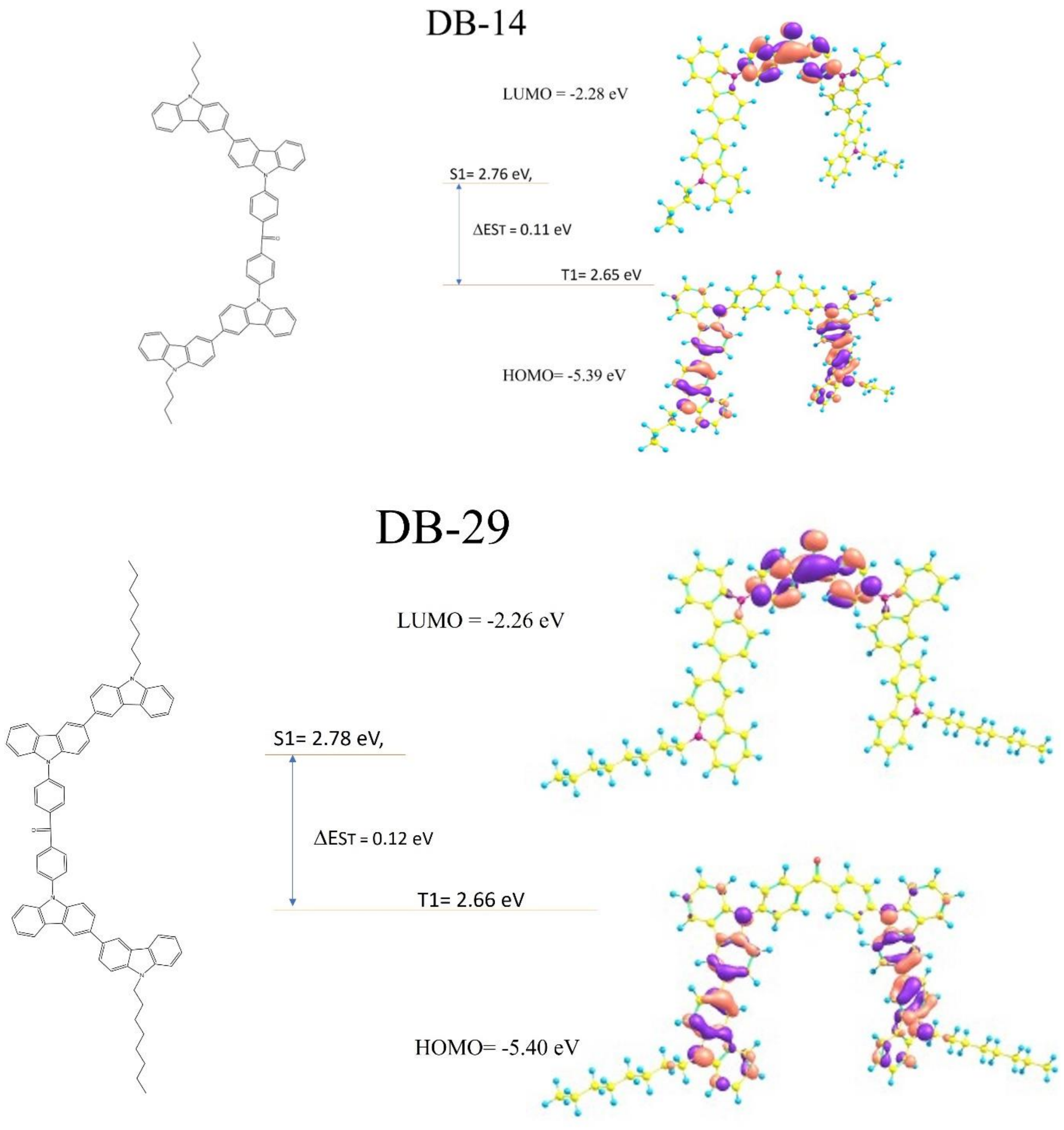

Figure S1. Electron density contours of FMO and HOMO, LUMO, singlet, triplet, and singlet-triplet energy gap electron distribution of the compounds **DB14** and **DB 29**.
